# Robust cytoplasmic accumulation of phosphorylated TDP-43 in transgenic models of tauopathy

**DOI:** 10.1007/s00401-013-1123-8

**Published:** 2013-05-11

**Authors:** Amy K. Clippinger, Simon D’Alton, Wen-Lang Lin, Tania F. Gendron, John Howard, David R. Borchelt, Ashley Cannon, Yari Carlomagno, Paramita Chakrabarty, Casey Cook, Todd E. Golde, Yona Levites, Laura Ranum, Patrick J. Schultheis, Guilian Xu, Leonard Petrucelli, Naruhiko Sahara, Dennis W. Dickson, Benoit Giasson, Jada Lewis

**Affiliations:** 1 Department of Neuroscience, Center for Translational Research in Neurodegenerative Disease, McKnight Brain Institute, University of Florida, 1275 Center Drive, BMS Building J-483, PO Box 100159, Gainesville, FL 32610-0244 USA; 2 Department of Biological Sciences, Northern Kentucky University, Highland Heights, KY 41099 USA; 3 Department of Neuroscience, Mayo Clinic, Jacksonville, FL 32224 USA; 4 SantaFe HealthCare Alzheimer’s Disease Center, Gainesville, FL USA; 5 Department of Molecular Genetics and Microbiology, Center for NeuroGenetics, College of Medicine, Genetics Institute, University of Florida, Gainesville, FL 32610 USA

**Keywords:** Tau, TDP-43, Mouse, Transgenic, Neuropathology, tauopathy, TDP-43 proteinopathies

## Abstract

Frontotemporal lobar degeneration (FTLD) has been subdivided based on the main pathology found in the brains of affected individuals. When the primary pathology is aggregated, hyperphosphorylated tau, the pathological diagnosis is FTLD-tau. When the primary pathology is cytoplasmic and/or nuclear aggregates of phosphorylated TAR-DNA-binding protein (TDP-43), the pathological diagnosis is FTLD-TDP. Notably, TDP-43 pathology can also occur in conjunction with a number of neurodegenerative disorders; however, unknown environmental and genetic factors may regulate this TDP-43 pathology. Using transgenic mouse models of several diseases of the central nervous system, we explored whether a primary proteinopathy might secondarily drive TDP-43 proteinopathy. We found abnormal, cytoplasmic accumulation of phosphorylated TDP-43 specifically in two tau transgenic models, but TDP-43 pathology was absent in mouse models of Aβ deposition, α-synucleinopathy or Huntington’s disease. Though tau pathology showed considerable overlap with cytoplasmic, phosphorylated TDP-43, tau pathology generally preceded TDP-43 pathology. Biochemical analysis confirmed the presence of TDP-43 abnormalities in the tau mice, which showed increased levels of high molecular weight, soluble TDP-43 and insoluble full-length and ~35 kD TDP-43. These data demonstrate that the neurodegenerative cascade associated with a primary tauopathy in tau transgenic mice can also promote TDP-43 abnormalities. These findings provide the first in vivo models to understand how TDP-43 pathology may arise as a secondary consequence of a primary proteinopathy.

**Electronic supplementary material:**

The online version of this article (doi:10.1007/s00401-013-1123-8) contains supplementary material, which is available to authorized users.

## Introduction

The major classes of frontotemporal lobar degeneration (FTLD) are those characterized by the presence of neuronal and glial inclusions composed of either tau protein (FTLD-tau) or TAR DNA-binding protein of 43 kDa (TDP-43; FTLD-TDP) [27, 40]. Familial forms of FTLD-tau are associated with mutations in the *MAPT* gene that encodes the tau protein, whilst mutations in *Granulin* (*GRN*), *Valosin Containing Protein* (*VCP*), or *C9ORF72* genes can cause FTLD-TDP or amyotrophic lateral sclerosis [3, 10, 22, 54]. Neurodegenerative conditions such as Alzheimer’s disease (AD), Huntington disease (HD), as well as Parkinson disease (PD) and dementia with Lewy bodies (DLB) are proposed to be “secondary” TDP-43 proteinopathies in which TDP-43 pathology occurs in the context of the distinctive hallmark pathology of each of these disorders [1, 21, 37, 47, 51]. Furthermore, TDP-43 pathology has been reported in the tauopathies argyrophilic grain disease [14] and corticobasal degeneration [51], but it is sparse in progressive supranuclear palsy [59]. The mechanistic connection between primary and secondary TDP-43 proteinopathies is unclear, but it is possibly related to unknown environmental or genetic factors.

One common feature in most human TDP-43 proteinopathies is the presence of cytoplasmic phosphorylated TDP-43 (pTDP-43), while normally TDP-43 is readily detected in the nucleus. Several studies have shown that antibodies specific for phosphorylated S403/404 and S409/410 TDP-43 recognize TDP-43 proteinopathies in humans [17, 38] and in transgenic mice overexpressing TDP-43 [7, 24, 57]. We sought to address the possible association between TDP-43 aggregation and other proteinopathies through the neuropathological analysis of mouse models of amyloidosis, tauopathy, α-synucleinopathy, and HD. This approach attempts to isolate the effect of each model’s defining genetic trigger and proteinopathy on TDP-43 aggregation, thereby eliminating parallel mechanisms that may cause TDP-43 pathology in humans (i.e., unrelated genetic or environmental factors). We discovered significant age-dependent accumulation of cytoplasmic, phosphorylated TDP-43 in two independent mouse models of tauopathy, but not in mouse models of amyloidosis, α-synucleinopathy, or (HD). As such, we demonstrate that tau-driven mechanisms can drive abnormal TDP-43 pathology in tau transgenic in vivo models.

## Materials and methods

### Transgenic mouse models

To study TDP-43 in the context of amyloidosis, we utilized TgCRND8 mice that overexpress a double mutant (K670N/M671L and V717F) form of human amyloid precursor protein (695 amino acid isoform—APP695) leading to age-dependent cognitive deficits and Aβ amyloid pathology [8]. We also studied Tg2576 mice that express human amyloid precursor protein with the Swedish double mutation K670N/M671I and that develop amyloid plaques [20] and Tg2576 mice crossed onto a P264L PS1 knock-in background that potentiates amyloid plaque formation [13, 48]. As models of tauopathy, we utilized the rTg4510 and the JNPL3 transgenic mouse models that express P301L (0N4R) human tau. The bigenic rTg4510 model uses a CaMKIIα-dependent tetracycline transactivator transgene [36] to drive the conditional expression of mutant human tau, and the resultant tauopathy is primarily found in the forebrain [46]. The JNPL3 mouse model utilizes the mouse prion promoter [5] to drive mutant human tau expression [31]. JNPL3 mice develop tauopathy in the spinal cord and hindbrain with less in forebrain, resulting in progressive motor dysfunction. M83 and M47 transgenic mouse models of α-synucleinopathy express A53T and E46K mutant forms of human α-synuclein, respectively, driven by the mouse prion protein promoter. These mice develop age-dependent severe motor impairments leading to death and widespread α-synuclein neuronal inclusions [11, 15]. To model HD, we utilized the N586-82Q-C63 mouse model expressing 586 amino acids of an N-terminal fragment of huntingtin containing 82 glutamine repeats, hereafter termed HD586-82Q. These mice develop robust cytoplasmic inclusions containing huntingtin [50].

### Antibodies

Anti-TAR-DNA-binding protein 43 (TDP-43) rabbit polyclonal antibody was purchased from ProteinTech Group (Chicago, IL). Rabbit anti-phospho Ser409/410 TDP-43 antibody is from CosmoBioUSA (Carlsbad, CA). Rabbit anti-phospho Ser410 TDP43 antibody is from Sigma-Aldrich (St. Louis, MO). pSer129 is a mouse monoclonal antibody that specifically recognizes phosphorylation of α-synuclein at S129 [55]. AT8 (Thermo-Fisher) is specific toward phosphorylation sites S202 and T205 in tau [16]. CP13 (provided by Dr. Peter Davies, Albert Einstein College of Medicine, New York, NY) recognizes phosphorylated tau at Ser202 site. PHF1 (generously provided by Dr. Peter Davies, Albert Einstein University, New York, NY) is specific towards phosphorylation sites S396 and S404 in tau [43]. AT100 (Thermo-Fisher) is specific toward phosphorylation sited S212 and T214 in tau. Anti-huntingtin mouse monoclonal antibody 2B4 (Millipore) was used to detect huntingtin aggregates. Anti-total Aβ antibody 33.1.1 was previously characterized [28]. Anti-β-actin rabbit polyclonal and anti-GAPDH rabbit polyclonal antibodies were purchased from Sigma-Aldrich (St. Louis, MO).

### Immunohistochemistry (IHC)

Mice were humanely euthanized. Fixed brains were paraffin embedded and sectioned. Intact spinal columns were immersion fixed, followed by fine dissection to remove vertebrae and subsequent post fixation. Sections were deparaffinized in xylene and rehydrated by immersion in a descending series of ethanols and steamed for 30 min. Peroxidase activity was quenched by incubation in an 80 % methanol/2 % H_2_0_2_ solution for 10 min. Sections were incubated with primary antibodies in 0.1 M Tris pH 7.6/2 % FBS overnight at 4 °C and subsequently incubated with biotinylated anti-rabbit or anti-mouse (Vector) for 1 h. To detect signal, a standard peroxidase ABC system (Vector) was used with a DAB reagent kit (KPL). Sections were counterstained with hematoxylin, rehydrated by an ascending series of ethanols and xylene, and cover slipped with Cytoseal (Thermo Scientific).

### Electron microscopy

Post-embedding immunogold electron microscopy (IEM) is essentially the same as previously described [33]. Rabbit polyclonal antibody to phosphorylated TDP-43 (S409/410) generated by LP was used. This antibody was produced by immunizing a rabbit with the peptide antigen CSMDSK[pS][pS]GWGM-COOH, representing amino acid residues 404–414 of full-length TDP-43 with S409 and S410 phosphorylation.

### Immunofluorescence staining

Sections were deparaffinized in xylene, rehydrated by immersion in a descending series of ethanols, steamed for 30 min, incubated with blocking solution (1 % fish skin gelatin/1 % BSA/2 % FBS/0.1 M Tris pH 7.5) for 2 h, incubated with primary antibodies in 0.1 % fish skin gelatin/5 % BSA/0.2 % FBS/0.1 M Tris pH 7.6 with azide overnight, and subsequently incubated with Alexa Fluor 594 goat anti-mouse and Alexa Fluor 488 goat anti-rabbit (Invitrogen) for 1 h. Sections were post-fixed with 10 % phosphate buffered formalin, immersed in amino-black to quench lipofuscin auto-fluorescence, and counterstained with DAPI (Pierce). Sections were mounted and cover slipped with Fluoromount G (Southern Biotech). Pictures were obtained using an Olympus BX51 fluorescent microscope with FITC, Texas Red, and DAPI filters. To visualize co-localization, images from each filter were layered in Photoshop.

### Western blotting

Sagittal half forebrains of rTg4510 and NT mice were frozen on dry ice and then fractionated as previously described [45]. 10 μg (soluble fraction, the protein concentration measured by BCA protein assay) or 10 μl (sarkosyl-insoluble fraction derived from 10 mg wet-weight of tissue) of protein was run on 10 % tris-glycine gel, transferred to nitrocellulose membrane and blocked in 5 % milk-TBST prior to probing with antibodies described above.

## Results

### Accumulation of phosphorylated, cytoplasmic TDP-43 in transgenic mouse models of tauopathies

Immunohistochemistry to visualize pTDP-43 (S409/410) was performed on mouse models of Aβ amyloidosis (TgCRND8, Tg2576, and Tg2576/P264L PS1), tauopathy (rTg4510 and JNPL3), α-synucleinopathy (Lines M47 and M83), HD (HD586-82Q), and non-transgenic (NT) controls at time points when each model has robust aggregates of their primary pathologic protein (e.g., tau in rTg4510 and α-synuclein in M83 mice). For rTg4510 mice, mice expressing the tTA activator in the absence of the tau responder were also used as a control.

**Fig. 1:**
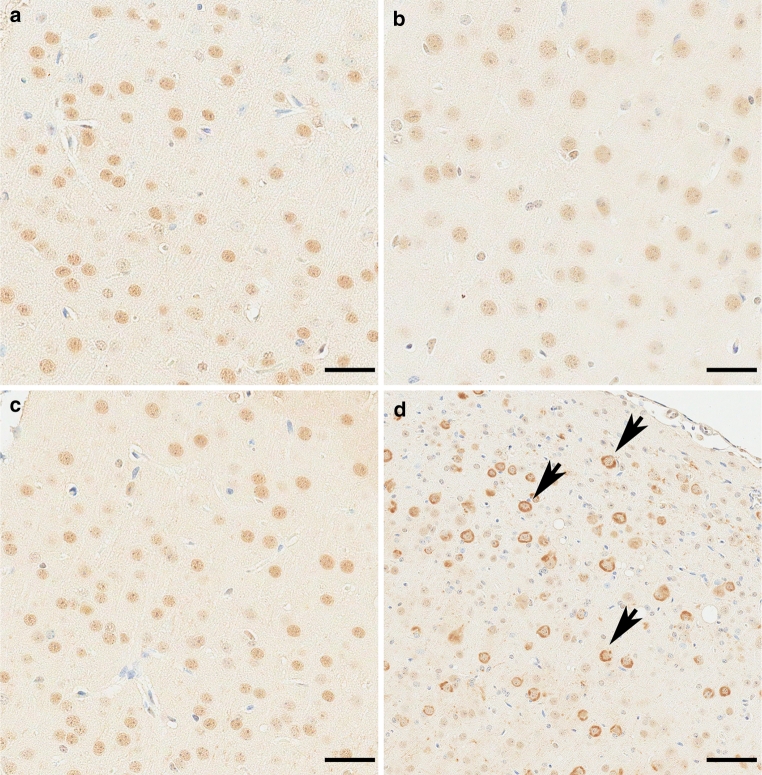
Phosphorylated TDP-43 progressively accumulates within the cytoplasm of forebrain neurons in the rTg4510 mouse model of tauopathy. Phosphorylated TDP-43 (pTDP-43; S409/410) is located within the nucleus in NT mice at 1.6 months (**a**) and tTA mice at 7.7 months (**b**). pTDP-43 is initial found in the nucleus of 1.6-month rTg4510 mice (**c**), and transitions into the cytoplasm of rTg4510 mice (**d**) by 7.8 months. Cortex is shown for all mice. *Bar* indicates 35 μm

In control mice, low levels of pTDP-43 (S409/410) were observed in the nucleus of some neurons (Fig. 1a, b). The normal nuclear localization of pTDP-43 is not altered by the presence of Aβ plaques in TgCRND8, Tg2576 and Tg2576/P264L PS1 mice, α-synuclein pathology in M47 and M83 mice or huntingtin inclusions in HD586-82Q mice (Electronic Supplementary Material 1; Supplementary Table 1). Normal nuclear localization of pTDP-43 was also observed in young rTg4510 mice (Fig. 1c). In contrast, we observed significant accumulation of pTDP-43 in the neuronal perikarya of older rTg4510 tau transgenic mice (Fig. 1d, arrows). The regional distribution of neurons with abnormal pTDP-43 cytoplasmic immunoreactivity was in multiple areas of the forebrain of the rTg4510 model, consistent with the region that normally develops robust tauopathy [46].

**Fig. 2:**
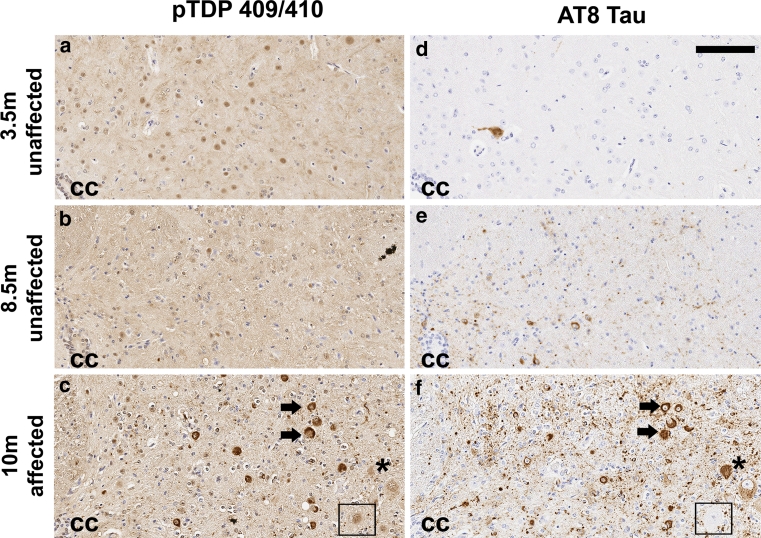
Tau pathology generally precedes the cytoplasmic accumulation of phosphorylated TDP-43 in the JNPL3 mouse model of tauopathy. Serial sections of spinal cord tissue from (**a**, **d**) 3.5, (**b**, **e**) 8.5 and (**c**, **f**) 10-month-old JNPL3 mice was immunostained for (**a**–**c**) TDP-43 phosphorylated at S409/410 and (**d**–**f**) tau phosphorylated at S202/T205 (AT8 antibody). JNPL3 mice at 3.5 months of age show (**a**) normal nuclear localization of pTDP-43 and (**d**) minimal tau pathology. (**b**) pTDP-43 remains localized in the neuronal nuclei as (**e**) tau pathology slowly accumulates in the spinal cord of 8.5-month-old JNPL3 lacking a motor phenotype. (**c**) Serial sectioning of a JNPL3 mouse with motor phenotype shows neurons with cytoplasmic relocalization of pTDP-43 (**c**, *arrows*) that also show prominent tau pathology (**f**, *arrows*). In addition, normal nuclear localization of pTDP-43 (**c**, *asterisk*) can be seen in cells with prominent tau pathology (**f**, *asterisk*). A healthy neuron without (**c**, *square*) cytoplasmic pTDP-43 or (**f**, *square*) tau pathology can also be seen. The central canal (*cc*) has been noted. The *bar* indicates 100 μm

To validate our results from the rTg4510 model and to begin to understand the mechanisms that lead to the abnormal cytoplasmic redistribution of phosphorylated TDP-43, we sought to determine the extent of overlap between TDP-43 and tau pathology, and the order in which it occurred. JNPL3 tau transgenic mice develop tauopathy in their spinal cord and have an age-progressive motor dysfunction [31]. The spinal cords of young and middle-aged JNPL3 mice that lacked motor dysfunction (Fig. 2a, b) showed normal nuclear localization of pTDP-43; however, the spinal cords of JNPL3 mice that had motor dysfunction showed striking cytoplasmic redistribution of pTDP-43 (Fig. 2c). We then immunostained serial sections of these animals with AT8 to visualize phosphorylated tau pathology (Fig. 2d–f). Initial tau pathology could be observed in young JNPL3 mice; however, striking tau pathology was not observed until JNPL3 mice developed motor dysfunction, similar to our previous reports [31, 60]. Using serial sections (Fig. 2c, f), we identified neurons containing both cytoplasmic pTDP-43 (Fig. 2c, arrows) and abnormal phosphorylated tau (Fig. 2f, arrows); neurons that had normal nuclear localization of pTDP-43 (Fig. 2c, asterisk) in the presence of tau pathology (Fig. 2f, asterisk); and neurons that had normal nuclear localization of pTDP-43 (Fig. 2c, square) without aggregated, phosphorylated tau protein (Fig. 2f, square). The majority of neuronal cell bodies with accumulations of pTDP-43 in the cytoplasm showed some degree of tau pathology (Fig. 2c, f). These data show that tau pathology can occur without the redistribution of pTDP-43 from the nucleus; however, pTDP-43 accumulation within the neuronal cytoplasm generally only occurs after tau pathology begins.

**Fig. 3:**
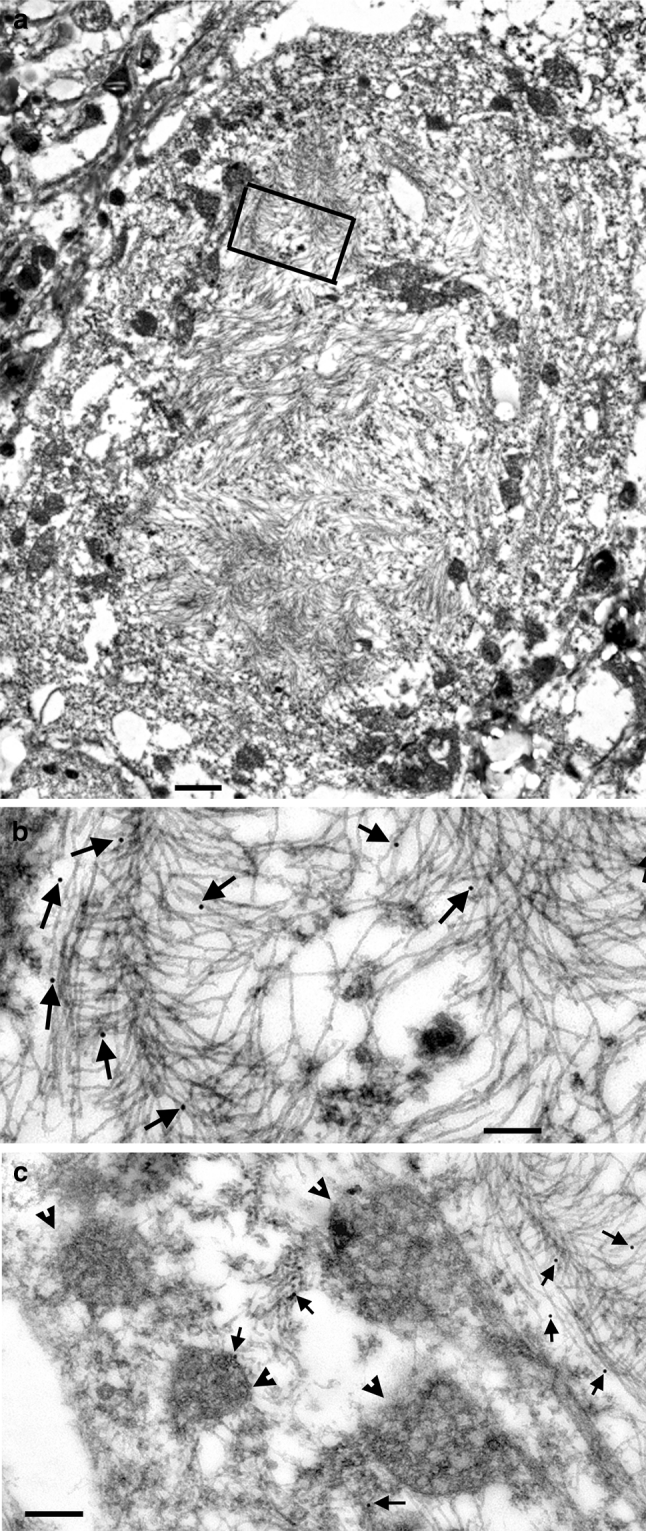
Immunogold EM demonstrates that TDP-43 is associated with tau fibrils in JNPL3 spinal cord neurons. Large filamentous aggregates in a spinal motor neuron of JNPL3 Tg mouse (**a**). Many mitochondria were pushed to the periphery. *Boxed area* is enlarged in the (**b**). *Bar*, 1 μm. Filamentous aggregates in herring-bone formation are labeled with pTDP-43 (**b**). *Arrows* point to gold particles. In the region directly adjacent to the *boxed area*, the mitochondria (*arrowheads*) serve as a marker of the area and there is no labeling on these and other organelles (**c**). Only few loose filaments are labeled (*arrows*). *Bar*, 0.2 μm

To confirm the association of phosphorylated TDP-43 with the tau aggregates, we performed ultrastructural analysis on spinal cord from a JNPL3 mouse with robust neurofibrillary tau pathology. As we have previously reported [34], tau filaments in the JNPL3 mice often assume a herring-bone order within the cell body (Fig. 3a, b). Gold labeling (Fig. 3b, arrows) indicated the presence of phosphorylated TDP-43 (S409/410) within the tau herring-bone structures, supporting our immunohistochemical data. Minimal gold particles were observed in regions of the neuron with low levels of tau filaments (Fig. 3c).

**Fig. 4:**
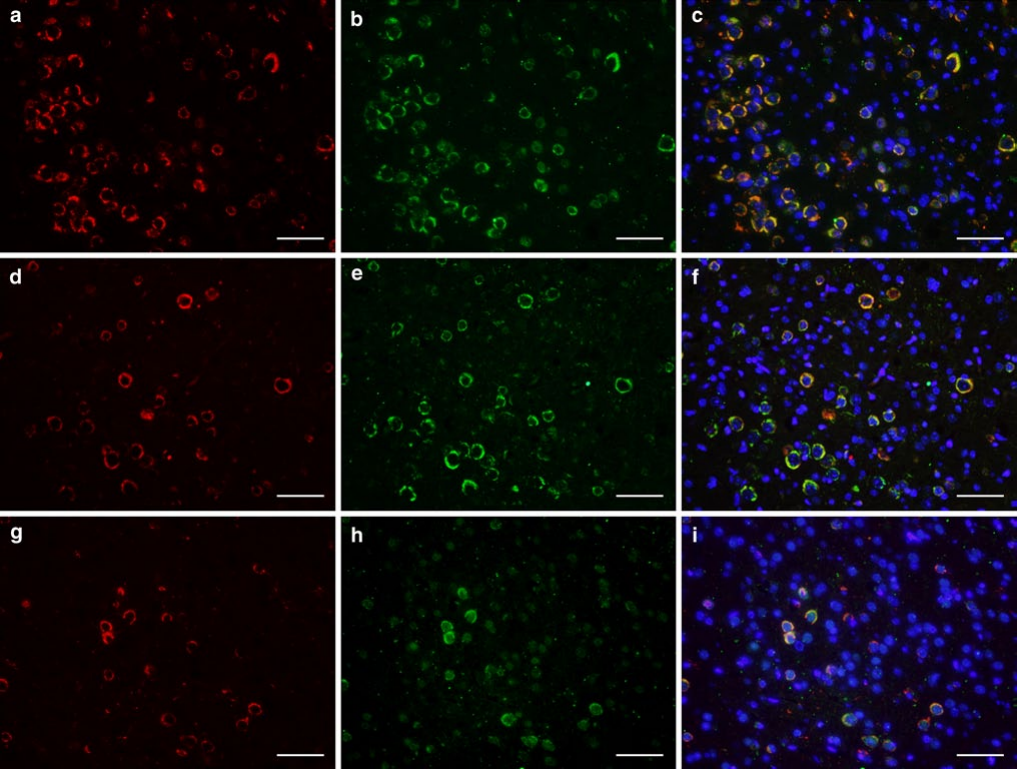
Cytoplasmic, phosphorylated TDP-43 (S409/410) co-localizes with tau pathology in cell bodies of the cortex of rTg4510 mice. Immunofluorescence shows pre-tangles and neurofibrillary tangles composed of hyperphosphorylated tau recognized by the antibody AT8 (**a**), AT100 (**d**), and PHF-1 (**g**) which co-localizes with cytoplasmic aggregation of pTDP-43, recognized by the S409/410 antibody (**b**, **e**, **h**; *green*). Co-localization between pTDP-43 (S409/410) and AT8 (**c**), AT100 (**f**), and PHF-1 (**i**) is shown in *yellow*. Nuclei were stained with DAPI (*blue*). Neurons shown are from the frontal cortex of an 8-month-old rTg4510 mouse at ×20 magnification. *White bar* indicates 50 μm

To determine the overlap between tau pathology and cytoplasmic accumulation of pTDP-43, we performed double immunofluorescence on rTg4510 brains with antibodies to hyperphosphorylated tau [(AT8; Fig. 4a, red), (AT100; Fig. 4d, red), (PHF1; Fig. 4g, red)] and phosphorylated TDP-43 (S409/410); Fig. 4b, e, h; green). Using this technique, we only observed cell body accumulation of pTDP-43 within neurons containing tau inclusions that were immunoreactive for hyperphosphorylated tau (Fig. 4c, f, i; yellow). Since the pTDP-43 (S409/410) antibody detects phosphorylation at a dual epitope, we sought to determine if an antibody that detected phosphorylation at only one of these epitopes (S410) would show similar overlap with phosphorylated tau. As with the dual phosphorylated TDP-43 epitope, there was a high degree of overlap between phosphorylated (S410) TDP-43 and phosphosphorylated tau (AT8, AT100, PHF-1) (Electronic Supplementary Material 2) in rTg4510 mice.

### TDP-43 is biochemically altered in rTg4510 mice

**Fig. 5:**
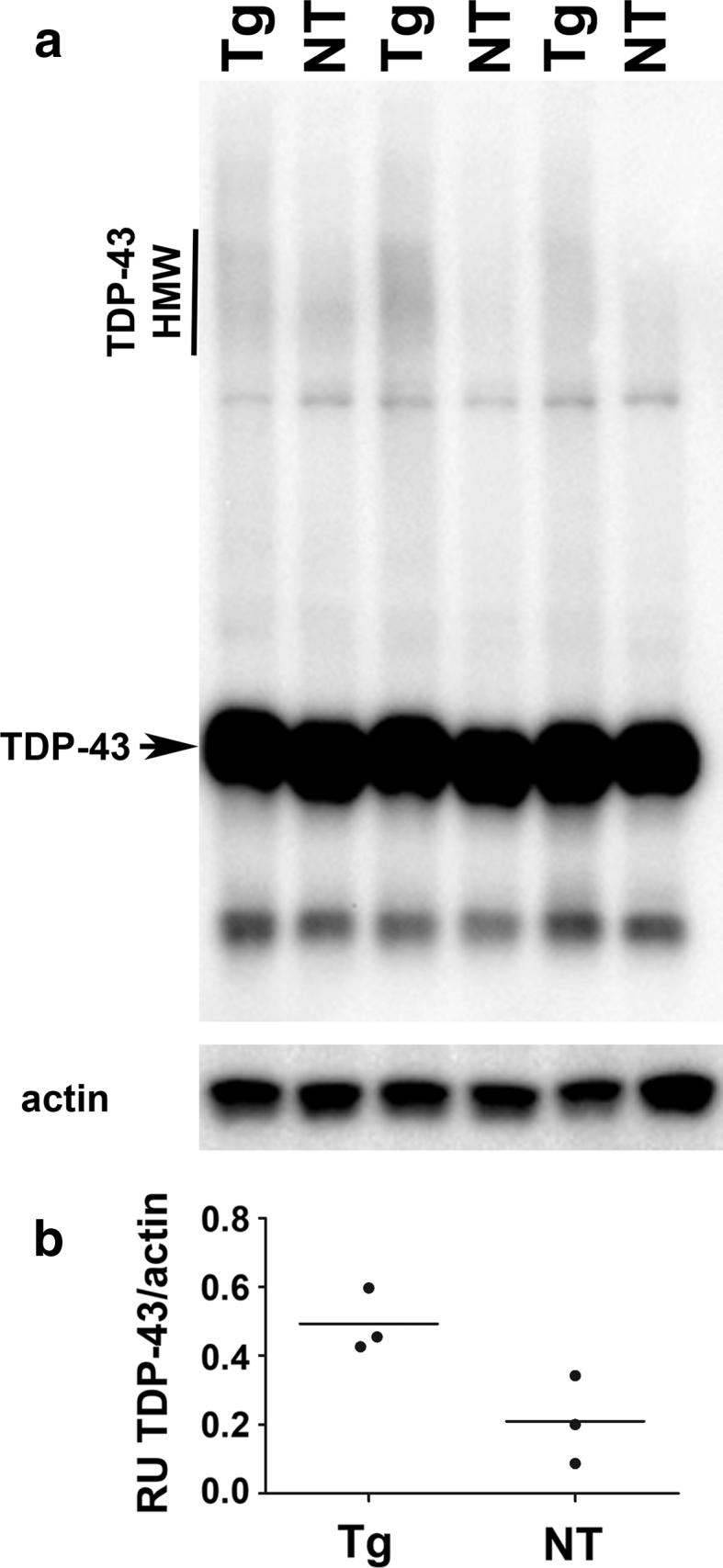
Higher molecular weight species of TDP-43 is elevated in the soluble fraction of rTg4510 compared to non-transgenic mice. (**a**) 10-month-old rTg4510 (Tg) and non-transgenic (NT) mice have equivalent expression of full-length TDP-43 protein (*arrow*). (**a**, **b**) rTg4510 mice have increased levels of high molecular weight TDP-43 protein (*line*, TDP-43 HMW) in the soluble fraction compared to NT mice (*p* = 0.03, unpaired t-test). β-Actin was used as a loading control. RU stands for relative units

To validate immunohistochemical findings, we performed Western blot analysis of TDP-43 in soluble and sarkosyl-insoluble fractions of forebrains of 10-month-old rTg4510 mice, an age in which tau pathology is present, as well as of NT control mice. Within the soluble fractions, levels of full-length TDP-43 were equivalent between rTg4510 and NT mice (Fig. 5a, arrow). Interestingly, rTg4510 mice had a higher molecular weight smear that was immunoreactive for TDP-43 (HMW; ~120–170 kDa) (Fig. 5a). Levels of these HMW species were ~2.5-fold higher in rTg4510 compared to NT mice (*p* = 0.03; Fig. 5b). β-Actin was utilized as a loading control.

**Fig. 6:**
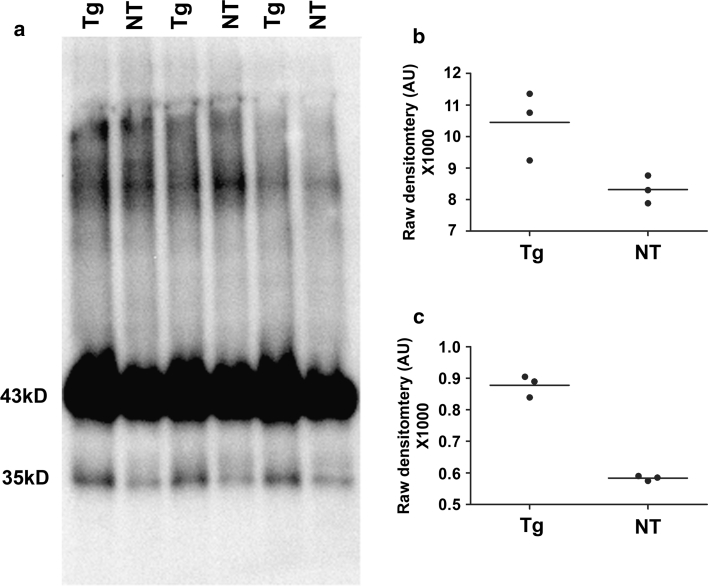
Full-length and ~35 kDa TDP-43 show increased insolublity in rTg4510 compared to NT mice. (**a**) 10-month-old rTg4510 (Tg) mice show significant increases in (**a**, **b**) full-length TDP-43 protein (*p* = 0.03) and (**a**, **c**) lower molecular weight ~35 kDa TDP-43, termed TDP-35 (*p* = 0.0001), within the sarkosyl-insoluble fraction compared to NT mice. AU stands for arbitrary units. Analyzed using unpaired t-test

As in human tauopathy, rTg4510 mice progressively accumulate hyperphosphorylated, aggregated tau within the detergent insoluble fraction (Electronic Supplementary Material 3) [46]. Given that tau and TDP-43 appeared to co-aggregate in a substantial proportion of forebrain neurons of rTg4510 mice, we sought to determine if rTg4510 mice contained elevated TDP-43 within the sarkosyl-insoluble fractions. We found an approximately 25 % increase in full-length 43 kDa TDP-43 in the sarkosyl-insoluble fraction from the forebrain of 10-month-old mice rTg4510 compared to NT mice (*p* = 0.03; Fig. 6a, b). Interestingly, we also observed an increase in ~35 kDa immunoreactive species of TDP-43, hereafter termed TDP-35, within the sarkosyl-insoluble fraction (*p* = 0.0001; Fig. 6a, c) of rTg4510 mouse forebrain. Although our *N* = 3 per genotype is low, these data were consistent and support our neuropathological analysis.

## Discussion

TDP-43 aggregation, cytoplasmic redistribution, phosphorylation and misprocessing characterize the pathology found in FTLD-TDP, associated with mutations in the *GRN*, *VCP*, and *C9ORF72* genes [3, 10, 54]. In contrast, a second form of frontotemporal lobar degeneration, FTLD-tau, is characterized by hyperphosphorylated, aggregated tau pathology, and many of the familial forms are caused by mutations in the *MAPT* gene that encodes tau protein [22]. Recently, Bieniek et al. [4] reported tau pathology in brains of individuals with FTLD associated with the *C9ORF72* expansion mutation, but similar elevation of tauopathy were not observed in FTLD associated with *GRN* mutations suggesting that an overlap of FTLD-TDP and FTLD-Tau may occur in the context of *C9ORF72*. Interestingly, King et al. [29] reported an individual with an A239T sequence variant in the *MAPT* gene as well as the *C9ORF72* expansion. This individual presented with dominant Pick-like tau pathology as well as the TDP-43 and p62 pathology that characterizes *C9ORF72* carriers; however, her siblings lacked this tau variant and developed typical pathology associated with the *C9ORF72* hexanucleotide repeat. Surprisingly, we were unable to find publications in which the authors clearly screened FTLD-tau cases with known pathogenic *MAPT* mutations for the level and/or distribution of phosphorylated TDP-43. TDP-43 pathology has been identified in a subset of different proteinopathies including tauopathies that occur in the absence of *MAPT* mutations [2, 19, 37]. The importance of this pathological overlap has been unclear. In the current study, we sought to utilize mouse models of Aβ amyloidosis, tauopathy, α-synucleinopathy, and a polyglutamine disorder (HD) to determine if any aspect of TDP-43 pathology can be driven in vivo by an independent primary pathological aggregate (e.g., tau) caused by a defined genetic event (e.g., mutant tau).

In healthy neurons, TDP-43 is primarily localized within the nucleus, and the redistribution and aggregation of TDP-43 within the cytoplasm are thought to be critical events in TDP-43-proteinopathies [40]. Much of the TDP-43 found within these aggregates is phosphorylated at serine residues (409/410) [38]. In the current study, we demonstrated the association of pTDP-43 within the cytoplasm of neurons burdened with pathological tau aggregates—a tauopathy triggered solely by the expression of mutant human tau in transgenic mice. These findings are consistent with a recent report showing the partial cytoplasmic redistribution of TDP-43 in JNPL3 mice during the course of tau inclusion formation using a non-phospho-specific TDP-43 antibody, although they did not show that this was directly associated with tau pathology [52].

In order to determine if the accumulation of pTDP-43 is directly related to the aggregation of hyperphosphorylated tau or simply the expression of high levels of human mutant tau protein, we examined brain and spinal cord from young rTg4510 and JNPL3 mice, respectively, with high levels of transgenic tau expression and minimal levels of hyperphosphorylated, aggregated tau protein. No change in TDP-43 phosphorylation or cytoplasmic distribution was observed in young tau mice. In contrast, cytoplasmic pTDP-43 was observed in both rTg4510 and JNPL3 mice after they developed overt tau pathology and, in the case of JNPL3, motor dysfunction. These results strongly support the idea that mutant tau expression alone does not induce pTDP-43 accumulation within the cytoplasm and that aggregation of tau is also critical.

The distribution of neurofibrillary tau pathology and neuronal loss in rTg4510 and JNPL3 mouse models of tauopathy closely correlated with the distribution of the neurons containing cytoplasmic accumulations of pTDP-43 protein. Hyperphosphorylated tau and pTDP-43 protein co-localized within many of the affected neurons; however, the overlap was incomplete since hyperphosphorylated tau aggregates could be found in some neurons in the absence of cytoplasmic pTDP-43. Rarely, the converse was observed. This data suggests that tau pathology precedes the redistribution of TDP-43 into the neuronal cytoplasm. Interestingly, pTDP-43 was present within the neurites of rTg4510 mice in the absence of AT8 immunopositive neuritic tau, suggesting that TDP-43 cytoplasmic accumulation may develop in other cellular domains after cytoplasmic redistribution is initiated by tau aggregation or other factors in the perikarya. Another interpretation is that neuritic pTDP-43 may co-localize with tau that is not phosphorylated at S202 or S202/T205, the phospho-tau epitopes recognized by CP13 and AT8 in the current study, respectively. Since most remaining cortical and hippocampal (CA1) neurons in the rTg4510 mice examined had tau pathology within the neuronal cell body, it seems likely that the pTDP-43 positive neurites originate from these affected cells; however, the methods utilized in this paper cannot exclude other origins of pTDP-43 localization within the neurites. Nevertheless, tau is a microtubule binding and stabilizing protein and it is possible that its aggregation perturbs normal microtubule function leading to the cytoplasmic accumulation of TDP-43 that might normally be transported to the nucleus or the presynaptic domains [35]. The notion that perturbation of microtubule function can lead to this cytoplasmic redistribution of TDP-43 is consistent with the observation of TDP-43 pathology in Perry syndrome, a rare parkinsonian disorder [56]. Perry syndrome is caused by mutations in *DCTN1*, the large p150^glued^ subunit of the dynactin complex [12] and cell culture studies show that the disruption of dynein-mediated microtubule transport can promote TDP-43 cytoplasmic aggregation [44].

To determine if the association between pTDP-43 and hyperphosphorylated tau altered the biochemical profile of TDP-43, we performed protein fractionation from brains of rTg4510 tau transgenic and NT mice. There was no change in total levels of TDP-43 in the soluble tau fraction across genotypes; however, rTg4510 mice did have a significant increase in a higher molecular weight smear that was immunopositive for TDP-43. The nature of these high molecular weight species is unclear; however, similar high molecular weight species have been identified in affected human brains although these tend to be found in insoluble, not soluble, fractions [1, 25, 39, 40]. It is unlikely that the TDP-43 in the HMW species is aggregated since it was localized in the soluble fraction, but it could reflect various post-translational modifications such as ubiquitination or oxidative modifications [9, 40]. Given that tau in human tauopathy and in the tau transgenic mice utilized in this study becomes hyperphosphorylated and aggregated, thereby shifting into the detergent insoluble fraction, we sought to determine if TDP-43 similarly shifted into the sarkosyl-insoluble fraction in association with the tauopathy in rTg4510. Indeed, we saw a significant increase of full-length TDP-43 within the sarkosyl-insoluble fraction when compared to NT mice, supporting the close association between the tau pathology observed in these mice and the cytoplasmic accumulation of pTDP-43. Intriguingly, we also observed a significant increase in a low molecular weight species of TDP-43 which we termed TDP-35 for its migration of ~35 kDa. The exact nature of TDP-35 and its relevance to the tauopathy observed in our models is unclear. In humans, it has been suggested that a similar 35 kDa species observed in TDP-43 proteinopathies may be generated from alternative translational or splicing pathways or may be the result of cleavage by caspase activity [41, 53, 61]. Indeed, caspase activation is a feature of the mouse models of tauopathy utilized in this study [49, 60].

The association between cytoplasmic pTDP-43 and tau appears specific since we saw no evidence of cytoplasmic relocalization of pTDP-43 in mouse models of Aβ amyloidosis, α-synucleinopathy or polyglutamine disease (HD), regardless of the broad spectrum of ages and stages of primary proteinopathy examined. A number of papers report TDP-43 pathology in AD with estimates ranging from 23 to 56 % [1, 2, 19, 21, 30, 51]. Many of the inclusions in human brains display close overlap between tau and TDP-43 [1, 2], similar to that observed in the tauopathy mice here. Lin and Dickson [32] also previously reported that in human AD brains, TDP-43 also can associate with tau within neuronal inclusions at the ultrastructural level.

The amyloid models that we utilized in the current study do not develop tauopathy similar to that observed in AD; therefore, it is possible that amyloidosis and tauopathy act in concert in AD to produce TDP-43 pathology. Caccamo et al. [6] reported that the 3XTg-AD amyloid model [42], which express mutant amyloid precursor protein, mutant presenilin 1, and P301L mutant tau protein, have increased full-length and ~35 kDa TDP in the low salt fraction and cytosolic fraction at 6 months of age, but not at 2 months or 12 months. Caccamo et al. [6] suggested that high levels of soluble amyloid beta oligomers positively correlated with TDP-43 changes, but they did not report an association with tau. Since the tauopathy in the 3XTg-AD model is much later and more modest than the amyloid pathology, it is not clear if the impact of tauopathy would have been observed by Caccamo et al. in the ages of mice examined. Herman et al. [18] also reported increased TDP-43 expression, cleavage and aggregation in association with intracellular amyloid beta 1–42 using lentiviral expression of amyloid beta 1–42 in rat motor cortex. Neither study reported an association of TDP-43 with the extracellular plaques that we examined in this report.

TDP-43 pathology is frequently observed in the brains (18–60 %) of patients with DLB [2, 19, 37], however, tau, α-synuclein and Aβ amyloid deposits often coexist in these brains making it difficult to assess which of these primary insults may trigger TDP-43 inclusion formation. In the current study, we used transgenic mice that primarily develop each specific type of these three inclusions to provide a useful indication of which one is more likely to contribute to TDP-43 cytoplasmic aggregation. The association between tau aggregation and pTDP-43 cytoplasmic aggregation in these tau transgenic mice could suggest that tau is the most critical factor driving TDP-43 aggregation in human DLB. Alternatively, that the co-occurrence of α-synuclein, tau and amyloid pathology in DLB could trigger an alternative mechanism which drives TDP-43 aggregation. Our currently available mouse models would not allow us to explore this scenario.

Our group has also shown that constitutive overexpression of wild-type and less so mutant TDP-43 can cause aggregation of hyperphosphorylated tau protein at S202, one of the two epitopes that is recognized by the AT8 antibody used in the current study [58]. This data also suggested that activation of PKC in the TDP-43 mice led to the hyperphosphorylated tau providing another link between tau and TDP-43. More recently, Jinwal et al. [26], reported that clearance of TDP-43 protein via a Cdc37/Hsp90 complex is impaired by the accumulation of tau. This recent finding also could underlie our in vivo findings in the tau transgenic mice that show robust aggregation of hyperphosphorylated tau protein.

Our results show a clear link between tau pathology and cytoplasmic accumulation of phosphorylated TDP-43 in the controlled in vivo systems of tau transgenic mice. It is currently unknown if this association between tau and TDP-43 can affect the disease course in either the mouse models or in human tauopathies. Our studies lay the groundwork for such investigations. Certainly, our data would suggest that groups with large cohorts of *MAPT* mutation carriers should assess their autopsy tissue for overlapping tau and TDP-43 pathology; however, such screens are generally precluded by the availability of tissue from known *MAPT* carriers. Functional studies which exploit the capabilities of the in vivo model systems utilized in this report could compliment these human studies. For example, the rTg4510 model of tauopathy can now be crossbred with conditional TDP-43 models created by our group and others [7, 24] to determine if the two pathologies act in concert to accelerate the FTLD-like neurodegeneration of these models. Furthermore, we can now suppress tau expression in the rTg4510 mice and determine if the TDP-43 pathology is reversible and if any reversion of TDP-43 pathology tracks with the cognitive recovery observed in tau suppressed rTg4510 mice [46]. In addition, seeding and spreading techniques [23] that are proving informative for the tau field could be expanded into TDP-43 transgenic mice (and vice versa) to help determine the cross talk between tau and TDP-43. Clarification of the disease relevance between tau and TDP-43 will ultimately allow us to determine if therapeutic efforts aimed at one molecule may hold promise against diseases characterized by the other protein.

## Electronic supplementary material

Below is the link to the electronic supplementary material.

Supplemental Figure 1. Neither extracellular amyloid plaques nor intracellular aggregates of α-synuclein and huntingtin cause accumulation of pTDP-43 within the cytoplasm of associated cells. Brains from 13 month old TgCRND8 amyloid precursor protein mice were immunostained with (a) anti-Aβ antibody 33.1.1 to visualize extracellular plaques and (b) anti-pTDP-43 (S409/410) antibody within the hippocampus. Hippocampus from 6 month old HD586-82Q huntingtin transgenic mice (labeled HD82) were immunostained with (c) anti-huntingtin antibody 2B4 and (d) anti-pTDP-43 (S409/410) antibody. Brain stem from 15 month old M47 α–synuclein transgenic mice were immunostained with (e) anti-pSer129 α-synuclein antibody to visualize α-synuclein pathology and (f) anti-pTDP-43 (S409/410) antibody. Brain stem from 15 month old M83 α–synuclein transgenic mice were immunostained with (g) anti-pSer129 α-synuclein antibody to visualize α-synuclein pathology and (h) anti-pTDP-43 (S409/410) antibody. Regions where the primary proteinopathy was robust were chosen for each model. The bars indicated 100 μm. Additional ages (Electronic Supplementary Table 1) for each model were examined and also found negative for cytoplasmic pTDP43 (409/410). Supplemental Figure 2: Cytoplasmic, phosphorylated TDP-43 (S410) co-localizes with tau pathology in cell bodies of the cortex of rTg4510 mice. Immunofluorescence shows pre-tangles and neurofibrillary tangles composed of hyperphosphorylated tau recognized by the antibody AT8 (a), AT100 (d), and PHF-1 (g) which co-localizes with cytoplasmic aggregation of pTDP-43, recognized by the S410 antibody (b, e, h; green). Co-localization between pTDP-43 (S410) and AT8 (c), AT100 (f), and PHF-1 (i) is shown in yellow and is similar to that observed between the dual phosphorylation TDP-43 epitope examined in Figure 4. Nuclei were stained with DAPI (blue). Neurons shown are from the frontal cortex of an 8 month rTg4510 mouse at 20X magnification. White bar indicates 50 μm. Supplemental Figure 3. rTg4510 show robust biochemical signatures of hyperphosphorylated, aggregated tau at 10 months of age. Soluble and sarkosyl-insoluble fractions from the saggital half forebrains of 10 month old rTg4510 (Tg) and NT mice were analyzed by Western blot using the CP13 antibody for phosphorylated tau (S202). These samples were the same fractions utilized for the biochemical analysis of soluble and insoluble TDP-43 levels (Figures 5 and 6). Human transgenic tau from rTg4510 mice shows a mobility shift from its normal MW at ~50kD into a 64kD species (arrow), representing the high degree of aggregation and hyperphosphorylation in these sample. Loading for soluble fractions was normalized to GAPDH (PDF 3853 kb).
