# Supplementary material for: Robust cytoplasmic accumulation of phosphorylated TDP-43 in transgenic models of tauopathy
Source: Acta Neuropathol. 2013 May 11;126(1):39–50. doi: 10.1007/s00401-013-1123-8 (PMC3690181; doi:10.1007/s00401-013-1123-8)
Supplement: Supplementary file 1 — Supplemental Figure 1. Neither extracellular amyloid plaques nor intracellular aggregates of α-synuclein and huntingtin cause accumulation of pTDP-43 within the cytoplasm of associated cells. Brains from 13 month old TgCRND8 amyloid precursor protein mice were immunostained with (a) anti-Aβ antibody 33.1.1 to visualize extracellular plaques and (b) anti-pTDP-43 (S409/410) antibody within the hippocampus. Hippocampus from 6 month old HD586-82Q huntingtin transgenic mice (labeled HD82) were immunostained with (c) anti-huntingtin antibody 2B4 and (d) anti-pTDP-43 (S409/410) antibody. Brain stem from 15 month old M47 α–synuclein transgenic mice were immunostained with (e) anti-pSer129 α-synuclein antibody to visualize α-synuclein pathology and (f) anti-pTDP-43 (S409/410) antibody. Brain stem from 15 month old M83 α–synuclein transgenic mice were immunostained with (g) anti-pSer129 α-synuclein antibody to visualize α-synuclein pathology and (h) anti-pTDP-43 (S409/410) antibody. Regions where the primary proteinopathy was robust were chosen for each model. The bars indicated 100 μm. Additional ages (Electronic Supplementary Table 1) for each model were examined and also found negative for cytoplasmic pTDP43 (409/410). Supplemental Figure 2: Cytoplasmic, phosphorylated TDP-43 (S410) co-localizes with tau pathology in cell bodies of the cortex of rTg4510 mice. Immunofluorescence shows pre-tangles and neurofibrillary tangles composed of hyperphosphorylated tau recognized by the antibody AT8 (a), AT100 (d), and PHF-1 (g) which co-localizes with cytoplasmic aggregation of pTDP-43, recognized by the S410 antibody (b, e, h; green). Co-localization between pTDP-43 (S410) and AT8 (c), AT100 (f), and PHF-1 (i) is shown in yellow and is similar to that observed between the dual phosphorylation TDP-43 epitope examined in Figure 4. Nuclei were stained with DAPI (blue). Neurons shown are from the frontal cortex of an 8 month rTg4510 mouse at 20X magnification. White bar indicates 50 μm [file 401_2013_1123_MOESM1_ESM.pdf]

Robust Cytoplasmic Accumulation of Phosphorylated TDP-43 in Transgenic Models of Tauopathy

Amy K. Clippinger[1, 2†], Simon D'Alton[1†], Wen-Lang Lin[3], Tania F. Gendron[3], John Howard[1], David Borchelt[1, 4], Ashley Cannon[3], Yari Carlomagno[3], Paramita Chakrabarty[1], Casey Cook[3], Todd E. Golde[1], Yona Levites[1], Laura Ranum[5], Patrick Schultheis[2], Guilian Xu[1], Leonard Petrucelli[3], Naruhiko Sahara[1], Dennis W. Dickson[3], Benoit Giasson[1‡*], Jada Lewis[1‡*]

[1]Department of Neuroscience, Center for Translational Research in Neurodegenerative Disease and McKnight Brain Institute, University of Florida, Gainesville, FL32610.

[2]Department of Biological Sciences, Northern Kentucky University, Highland Heights, KY 41099

[3]Department of Neuroscience, Mayo Clinic, Jacksonville, FL32223

[4]SantaFe HealthCare Alzheimer's Disease Center

[5]Center for NeuroGenetics, Department of Molecular Genetics and Microbiology and Genetics Institute, College of Medicine, University of Florida, Gainesville, FL 32610

Running title: TDP-43 pathology in tau transgenic mice

[†]These authors contributed equally

[‡]These authors contributed equally

***Address correspondence to:**

Dr. Benoit Giasson, Department of Neuroscience, Center for Translational Research in Neurodegenerative Disease, University of Florida, 1275 Center Drive, BMS Building J-483, PO Box 100159, Gainesville, FL32610-0244. Tel: 352-273-9363; E-mail:bgiasson@ufl.edu.

Dr. Jada Lewis, Department of Neuroscience, Center for Translational Research in Neurodegenerative Disease, University of Florida, 1275 Center Drive, BMS Building J-487, PO Box 100159, Gainesville, FL32610-0244. Tel: 352-273-9666; E-mail:jada.lewis@ufl.edu.

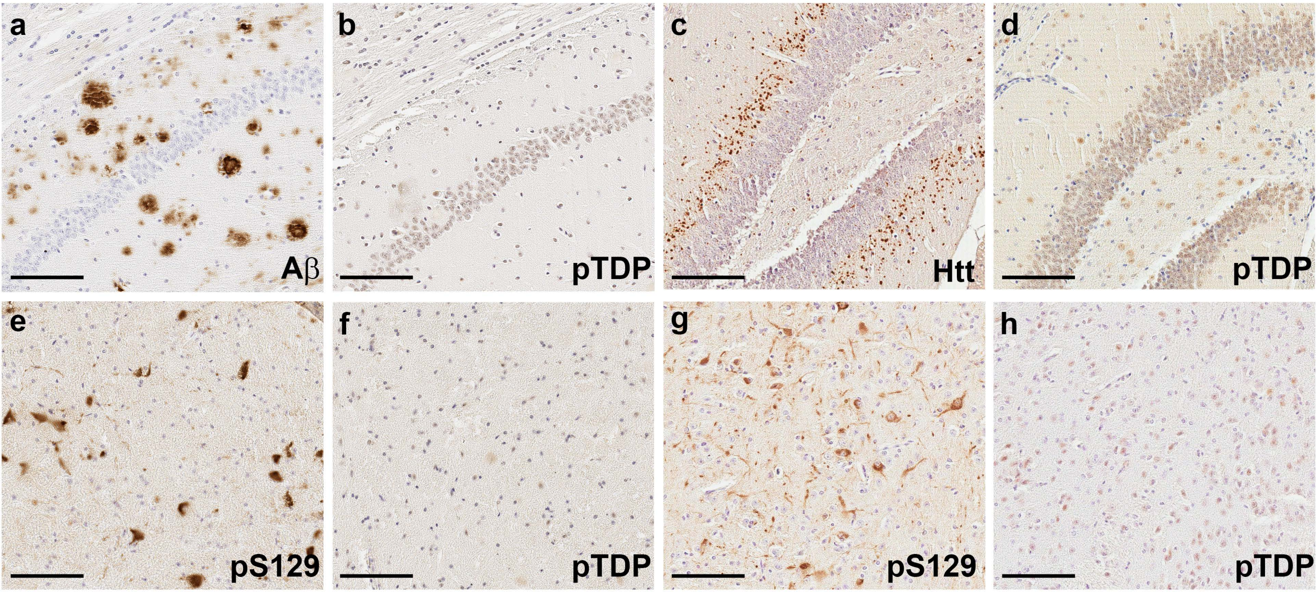

a
Aβ
b
pTDP
c
Htt
d
pTDP
e
pS129
f
pTDP
g
pS129
h
pTDP

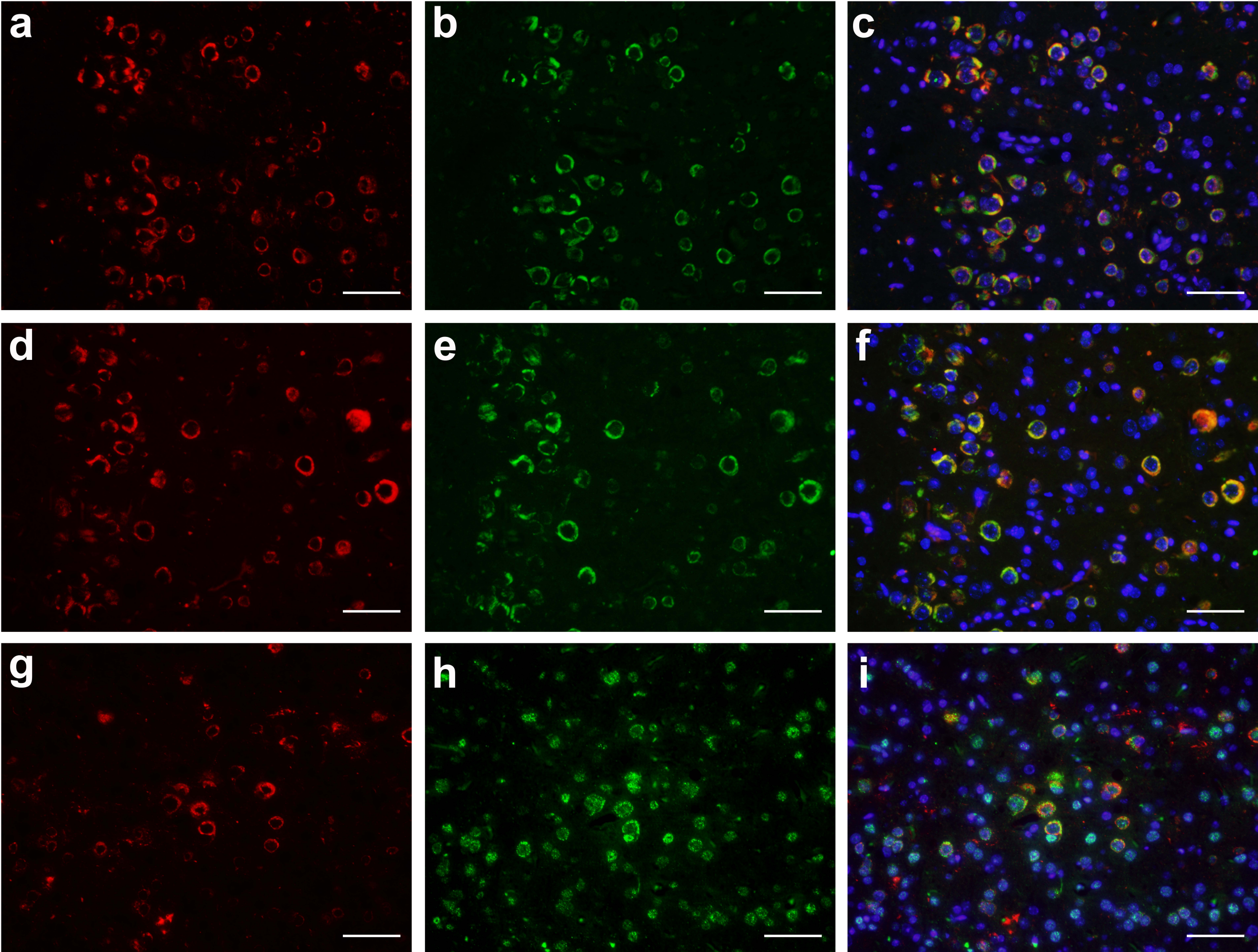
a
b
c
d
e
f
g
h
i

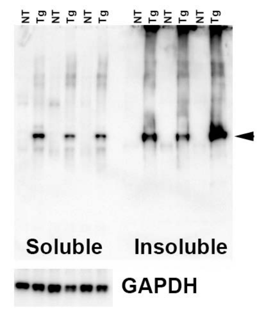
NT
Tg
NT
Tg
NT
Tg
NT
Tg
NT
Tg
NT
Tg
Soluble
Insoluble
GAPDH

Supplemental Table 1: Models that lacked TDP-43 (p409/410) cytoplasmic accumulation			
Model	Primary Pathology	Ages (months)	Total N
Tg2576	Amyloid beta plaques	9-26	11
CRND8		4-9	9
Tg2576/P246L PSEN1 KI		5-15	8
M47	Synucleinopathy	6-26	11
M83		4-15	11
N586-82Q-C63	Huntingtin aggregates	1-8	11
